# Toward Intelligent Rehabilitation Program Management: A System-Level Review of AI Architectures

**DOI:** 10.3390/bioengineering13050539

**Published:** 2026-05-07

**Authors:** Catalina Luca, Ilie Onu, Sardaru Dragos, Daniela Viorelia Matei, Robert Fuior, Calin Petru Corciova

**Affiliations:** Department of Biomedical Sciences, Faculty of Medical Bioengineering, “Grigore T. Popa” University of Medicine and Pharmacy, 700115 Iași, Romania; catalina.luca@umfiasi.ro (C.L.); ilie.onu@umfiasi.ro (I.O.); dragos.sardaru@umfiasi.ro (S.D.); daniela.matei@umfiasi.ro (D.V.M.); calin.corciova@umfiasi.ro (C.P.C.)

**Keywords:** clinical decision support systems, predictive analytics, computer vision, personalized therapy, digital health

## Abstract

Artificial intelligence (AI) is reshaping medical rehabilitation by advancing from isolated assistive technologies toward data-driven program management. Beyond established applications in robotics and virtual reality, AI enables multimodal data integration, predictive analytics, adaptive therapy optimization, and real-time monitoring across rehabilitation domains. This review synthesizes 61 peer-reviewed studies to examine how AI supports the management, planning, and evaluation of rehabilitation programs. The evidence indicates strong technical maturity at the device and session levels, particularly in robotic control and wearable monitoring, whereas longitudinal program orchestration and system-level coordination remain at an emerging stage. Machine learning, reinforcement learning, computer vision, and time-series models facilitate patient phenotyping, therapy personalization, and prognostic modeling. However, their scalability is constrained by limited interoperability, heterogeneous outcome measures, and insufficient multicenter validation. A structured six-layer management architecture is proposed to conceptualize AI as an integrated orchestration framework. Advancing toward scalable and trustworthy rehabilitation ecosystems will require interoperable infrastructures, longitudinal validation, and embedded ethical and explainability mechanisms.

## 1. Introduction

Rehabilitation represents a cornerstone of modern healthcare, aiming to restore functional independence and improve quality of life across a wide spectrum of neurological, orthopedic, cardiovascular, and developmental conditions. As global demand for rehabilitation services continues to increase, healthcare systems face mounting pressures related to workforce limitations, rising costs, and the need for personalized treatment strategies [1].

Over the past decade, artificial intelligence (AI) has emerged as a transformative technological force within healthcare. In rehabilitation medicine, AI has been applied to robotic gait training, computer vision-based motion tracking, predictive modeling of recovery, virtual reality-based therapy, and remote monitoring platforms [2]. These advances have demonstrated improvements in the accuracy, personalization, and accessibility of therapeutic interventions [3,4].

However, despite a rapidly expanding body of literature, most existing studies focus on isolated AI applications rather than the broader problem of rehabilitation program management. Most of the published work describes what individual AI systems can do, for example, predicting complications or adjusting robotic assistance, but rarely addresses how these systems integrate into a structured, longitudinal management framework [5]. As a result, the concept of “AI-driven rehabilitation management” remains ambiguously defined.

Effective rehabilitation management extends beyond device-level automation. It involves coordinated processes including patient stratification, therapy planning, real-time adaptation, longitudinal outcome monitoring, and resource allocation. Without a system-level perspective, AI risks remaining fragmented into disconnected technological solutions [6,7].

To address this conceptual gap, this review moves beyond a descriptive summary of AI tools and proposes a layered architectural framework for AI-driven rehabilitation program management. By synthesizing empirical findings across neurological, developmental, cardiovascular, and neurodegenerative domains, we reorganize the literature according to functional management layers rather than technological categories.

The scope of this review is to define rehabilitation management in operational, system-level terms. For this, in this article we propose a six-layer architecture integrating data acquisition, stratification, therapy optimization, adaptive monitoring, prognostic modeling, and institutional coordination.

Achieving this, we manage to differentiate device-level intelligence from program- and system-level orchestration. Also, we have been able to identify critical technical and translational gaps that must be addressed for scalable clinical implementation.

By reframing AI in rehabilitation as an orchestration engine rather than a collection of isolated technologies, this review aims to provide a structured foundation for the next generation of intelligent rehabilitation systems.

## 2. Methods

### 2.1. Study Design and Scope

This review was conducted following the Preferred Reporting Items for Systematic Reviews and Meta-Analyses (PRISMA 2020) guidelines [8]. The objective was to identify and synthesize peer-reviewed evidence concerning artificial intelligence applications specifically related to the management, planning, monitoring, and optimization of medical rehabilitation programs.

The review focused on rehabilitation domains in which AI demonstrates program-level or decision-support potential, including personalized rehabilitation systems, assistive robotics, neurological and developmental disorder rehabilitation, virtual and extended reality-based rehabilitation, neurodegenerative disease management, and cardiovascular telerehabilitation.

### 2.2. Search Strategy

A systematic search was conducted in four electronic databases: PubMed, Scopus, Web of Science and Google Scholar.

The search covered publications between 2010 and 2025 because this time frame showed how clinical artificial intelligence applications developed during their most active growth period.

Search terms were combined using Boolean [8] operators and adapted for each database. The core query structure included:

- (“Artificial Intelligence” OR “AI” OR “Machine Learning” OR “Deep Learning”).
- (“Medical Rehabilitation” OR “Physical Therapy” OR “Rehabilitation Programs” OR “Neurorehabilitation”).
- (“Management” OR “Planning” OR “Monitoring” OR “Optimization”).

Reference lists of included articles were also manually screened to identify additional relevant publications.

### 2.3. Eligibility Criteria

Studies were excluded if they:

- Were conference abstracts, editorials, commentaries, or opinion papers.
- Focused exclusively on AI algorithm development without application in clinical rehabilitation contexts.
- Addressed assistive technologies without demonstrating relevance to rehabilitation program management.
- Were not available in full-text form.
- Were not published in English.

### 2.4. Study Selection Process

The titles and abstracts of all records were independently screened by two reviewers. Any disagreements at this stage were resolved through discussion; when consensus could not be reached, a joint re-evaluation was conducted until agreement was achieved.

**Figure 1:**
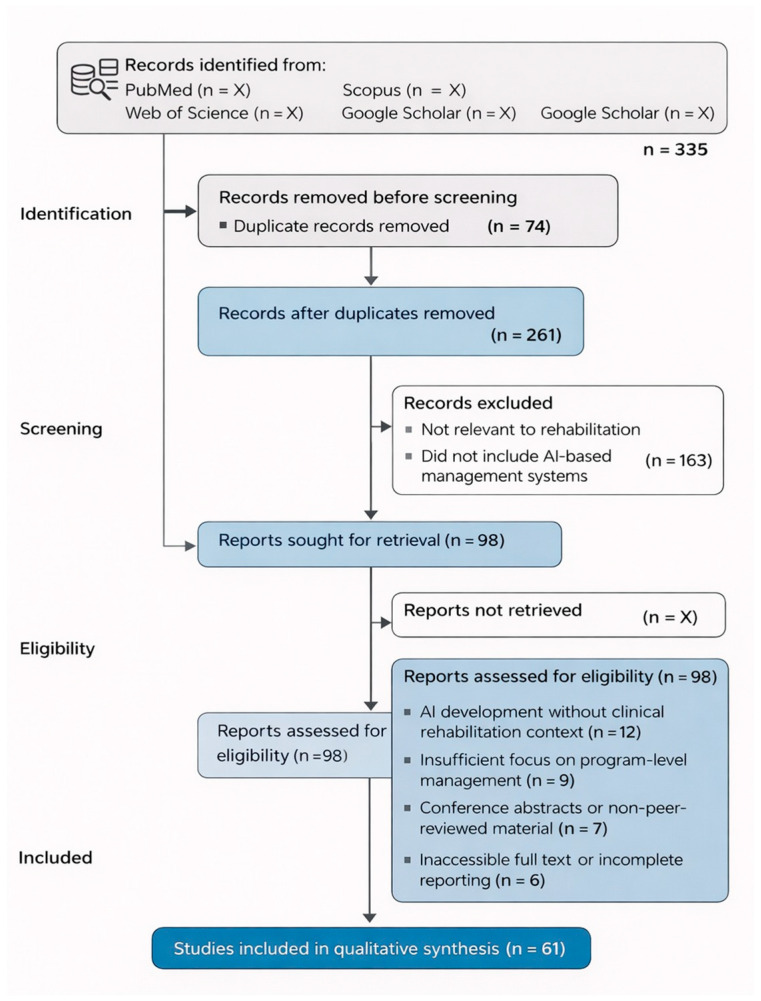
PRISMA 2020 flow diagram of the study selection process.

Full-text articles were subsequently assessed independently by the same reviewers for eligibility as can be seen in Figure 1. Discrepancies in inclusion decisions were resolved through discussion and consensus.

### 2.5. Data Extraction and Synthesis

Data were extracted using a standardized form developed prior to analysis. The following variables were recorded:

- Author(s) and year of publication.
- Study design.
- AI methodology (e.g., supervised learning, reinforcement learning, neural networks).
- Rehabilitation domain (e.g., stroke, cardiovascular, neurodevelopmental).
- Management layer addressed (e.g., stratification, therapy optimization, monitoring).
- Clinical validation level (pilot, observational, randomized trial).
- Reported outcomes and key findings.

Studies were synthesized qualitatively and categorized according to the layered rehabilitation management framework proposed in this review (data acquisition, stratification, planning, adaptation, prognostic modeling, and system coordination). A detailed summary of all included studies (n = 61), including rehabilitation domain, management layer, AI methodology, validation level, and reported effectiveness, is provided in Supplementary Files Table S1: [Included studies table auto-extracted from the manuscript reference list].

### 2.6. Risk of Bias Considerations

Given the heterogeneity of study designs and outcome measures, a formal meta-analysis was not conducted. Instead, methodological rigor was qualitatively assessed based on:

- Study design (observational vs. randomized controlled trial).
- Sample size.
- Duration of follow-up.
- External validation presence.
- Transparency of AI model reporting.

Most included studies demonstrated moderate methodological quality, with limited long-term validation and relatively small cohorts, particularly in program-level AI applications. Due to heterogeneity, no formal bias tool (e.g., ROBIS) was applied.

## 3. AI Applications Across Rehabilitation Domains

The characteristics of the included studies are summarized in Supplementary Materials, which provides a structured overview of the evidence base supporting the following analysis. Artificial intelligence is increasingly embedded across multiple rehabilitation domains, reshaping therapeutic design, delivery, and longitudinal monitoring. Rather than functioning as isolated technological adjuncts, AI systems increasingly support personalization, adaptive control, predictive modeling, and data-driven program optimization within diverse clinical contexts [9].

Randomized controlled evidence demonstrates that AI-enabled digital rehabilitation platforms can achieve clinical outcomes equivalent to conventional in-person physiotherapy, while substantially improving patient engagement and adherence metrics [10]. These findings underscore the feasibility of scalable, digitally mediated rehabilitation pathways without compromising therapeutic efficacy.

In parallel, advances in adaptive control frameworks have expanded the clinical relevance of wearable robotic systems. Human-in-the-loop optimization strategies for soft exoskeletons have shown significant improvements in gait efficiency among individuals with spinal cord injuries, highlighting the capacity of AI-driven assistive technologies to dynamically modulate biomechanical support in response to user performance [11]. Such developments exemplify the transition from static assistance devices to responsive, learning-enabled rehabilitation systems.

Wearable sensors and mobile health platforms further reinforce this paradigm shift by enabling continuous, ecologically valid monitoring of motor performance. The acquisition of high-resolution movement and physiological data in real-world settings enhances impairment detection, refines functional assessment, and facilitates timely therapeutic adjustment [12,13]. These capabilities contribute to a broader transformation from episodic, clinic-centered care toward continuously optimized rehabilitation trajectories.

Collectively, the contemporary literature demonstrates that AI applications extend across personalized rehabilitation systems, robotic-assisted interventions, neurological and developmental rehabilitation, immersive virtual and extended reality environments, neurodegenerative disease management, and cardiovascular telerehabilitation [14,15]. The following sections synthesize these domain-specific implementations while situating them within a structured program-management perspective.

### 3.1. Robotics and AI-Enabled Wearable Systems

Robotic-assisted rehabilitation remains one of the most technically mature domains of AI integration. Exoskeletons and cable-driven robotic platforms increasingly incorporate adaptive control algorithms that dynamically modulate assistance based on biomechanical and kinematic feedback [11,16]. Human-in-the-loop optimization strategies have demonstrated significant improvements in gait efficiency in individuals with spinal cord injury and post-stroke impairments [11].

Recent developments in personalized robotic telerehabilitation further expand these capabilities. Huang et al. demonstrated that AI-integrated robotic platforms enable remote adaptive modulation of assistance levels using real-time sensor fusion and reinforcement learning approaches, improving functional training precision in home-based settings [15]. An AI-integrated exoskeleton with fingertip haptic stimulation improved motor recovery and engagement in stroke rehabilitation, while a randomized trial of the Ekso GT^®^ in patients with Parkinson’s disease showed enhanced gait stability and reduced freezing episodes through real-time adaptive analysis [17].

Wearable systems equipped with inertial measurement units (IMUs), electromyography sensors, and machine learning classifiers provide continuous motion analysis and motor intent detection [18]. These systems facilitate closed-loop adjustment during therapy sessions and contribute to data-informed personalization. However, despite strong device-level validation, most robotic systems remain confined to session-level adaptation rather than integration into longitudinal rehabilitation program management architectures [12,19].

### 3.2. Neurological and Developmental Rehabilitation

Between 2020 and 2025, AI applications in neurological rehabilitation expanded considerably. Machine learning models are increasingly applied for neuroimaging interpretation, functional outcome prediction, and patient stratification in stroke, spinal cord injury, and Parkinson’s disease [20,21]. These approaches improve diagnostic precision and enable risk-adjusted therapy planning.

Reinforcement learning-based precision rehabilitation models represent a notable advancement in therapy optimization. Ye et al. proposed a contextual reinforcement learning framework capable of adapting rehabilitation intensity based on patient performance trajectories, demonstrating the feasibility of longitudinal AI-driven dosage optimization [22]. Such systems illustrate the shift from reactive therapy adjustment toward predictive and adaptive management.

In developmental rehabilitation, AI-driven digital phenotyping platforms enable early identification of language and executive function disorders [13,23]. Broader analyses of AI-based pediatric assessment platforms indicate that automated, scalable screening systems significantly improve accessibility while maintaining diagnostic reliability [24].

Tele-assessment tools and remote executive function monitoring platforms further expanded during the COVID-19 period [25], reinforcing the role of AI in continuity of care. However, despite growing diagnostic sophistication, integration with full program orchestration remains limited.

### 3.3. AI-Enhanced Virtual and Extended Reality Systems

Virtual and extended reality platforms have become central to immersive rehabilitation environments. When integrated with AI, VR/XR systems adapt task complexity dynamically, generate synthetic performance datasets, and provide granular analytics of motor and cognitive performance [13].

Systematic reviews confirm that VR-based interventions significantly improve upper-limb motor recovery and functional independence compared with conventional therapy [14]. AI integration enhances these systems by enabling real-time difficulty modulation and predictive performance tracking [26].

AI-driven virtual reality (VR) systems provide immersive motor training environments in which patients perform repetitive, task-oriented movements with real-time feedback and adaptive difficulty modulation, as exemplified by platforms such as NEUROFORMA (Titanis) [27].

AI-powered serious games, including platforms such as Lumosity and Brain Age, provide adaptive cognitive training across attention, memory, and executive functions by dynamically adjusting task difficulty to maintain optimal engagement [28,29].

Head-mounted displays (HMDs) used in VR-based rehabilitation immerse patients in interactive therapeutic environments for assessment and intervention in conditions such as stroke, multiple sclerosis, spinal cord injury, and Parkinson’s disease, with AI dynamically adjusting task complexity according to patient performance [30].

Interactive VR gloves integrate motion tracking and haptic feedback to enable patients to manipulate virtual objects and perform targeted rehabilitative hand exercises [31].

El-Banna et al. emphasized that AI-enhanced VR environments increasingly function as adaptive therapeutic ecosystems rather than static interactive tools, enabling personalized progression modeling and integrated monitoring [14]. Furthermore, Jleli et al. described AI-driven remote monitoring models capable of synchronizing VR task analytics with wearable sensor streams, facilitating real-time therapeutic adjustment in remote settings [32].

Despite strong engagement outcomes and feasibility [33], most VR systems operate within isolated therapy sessions. Interoperability with institutional management systems remains an emerging research direction.

### 3.4. Neurodegenerative Disease Rehabilitation

AI applications in neurodegenerative disorders focus on early detection, progression modeling, and cognitive training optimization. Machine learning models applied to structural MRI and demographic datasets support early risk prediction for Alzheimer’s disease and Parkinson’s disease [34,35]. Predictive modeling allows stratified prognosis estimation and supports preventive intervention strategies.

Personalized cognitive rehabilitation platforms employ adaptive algorithms that continuously adjust difficulty based on neuropsychological performance [36,37]. AI-enabled telerehabilitation systems further extend monitoring capabilities into home environments, enhancing longitudinal management [38]. AI applications in speech and motor rehabilitation involve using machine learning algorithms to improve speech recognition and motor function. AI systems can provide real-time feedback and adjust therapy exercises to improve patient outcomes [39]. Studies have shown that AI can significantly enhance the rehabilitation process by providing precise and timely interventions [40].

Functional Electrical Stimulation (FES) uses AI-driven control of electrical impulses to activate targeted muscle contractions, thereby facilitating motor function recovery [38].

Recent analyses underscore that AI-based digital biomarkers derived from longitudinal datasets may improve early disease trajectory estimation and therapy personalization [24]. However, most predictive studies rely on retrospective datasets and require multicenter prospective validation [41].

### 3.5. Cardiovascular Telerehabilitation

Cardiovascular telerehabilitation (CTR) represents a structured example of AI-supported remote program delivery. AI-driven platforms analyze physiological parameters, adherence metrics, and digital biomarkers to personalize exercise regimens and modulate intensity safely [42,43].

Blended AI–human supervision models demonstrate moderate improvements in cardiovascular risk profiles and sustained physical activity levels [44]. AI-generated digital biomarkers further support risk stratification and early detection of adverse trends.

AI-driven telerehabilitation platforms monitor cardiovascular parameters during home-based exercise and dynamically adjust intensity and modality in real time, as exemplified by the REHAB+ mobile cardiac rehabilitation program designed to enhance accessibility and long-term adherence [45].

AI-driven aerobic and resistance training programs personalize exercise prescriptions for cardiovascular rehabilitation by continuously adjusting intensity and content based on patient performance and feedback [46].

Recent system-level analyses indicate that AI-supported remote monitoring infrastructures may enable scalable cardiovascular management models if integrated with institutional scheduling and risk prediction frameworks [14,32]. However, long-term outcome validation and protocol standardization remain limited.

### 3.6. Cross-Domain Synthesis

Across domains, AI integration demonstrates strongest maturity in:

✓ Multimodal data acquisition and wearable monitoring.
✓ Adaptive session-level control.
✓ Predictive risk modeling.

Emerging but less mature areas include:

✓ Reinforcement learning-based therapy optimization [22].
✓ Cross-platform remote monitoring architectures [32].
✓ Integrated VR-based adaptive ecosystems [14].
✓ Longitudinal digital biomarker-driven prognosis [24].

**Figure 2:**
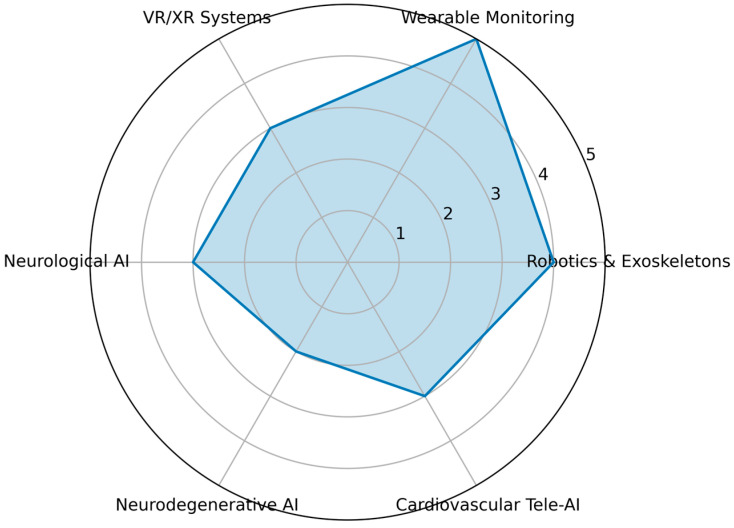
Translational maturity of AI integration across rehabilitation domains.

Figure 2 (radar plot) illustrates this uneven translational maturity, highlighting that robotics and wearable systems show higher deployment readiness, whereas neurodegenerative prognostic modeling and cardiovascular program orchestration remain developing areas.

Collectively, the state-of-the-art demonstrates a transition from isolated AI-assisted tools toward coordinated rehabilitation management platforms [47].

However, widespread implementation will require interoperable data infrastructures, multicenter validation of adaptive learning models, and embedded ethical governance mechanisms.

## 4. A System-Level Architecture for AI-Driven Rehabilitation Program Management

While numerous studies describe the use of artificial intelligence in isolated rehabilitation tasks, such as gait correction, robotic assistance, or outcome prediction, there remains a lack of conceptual clarity regarding how AI functions at the program management level (see Table 1). To address this gap, we propose a system-level architecture model for AI-driven rehabilitation program management that distinguishes between device-level intelligence and program-level orchestration [48].

Rehabilitation management is not limited to exercise execution [49]. It encompasses patient stratification, therapy planning, adaptive optimization, outcome evaluation, and resource coordination. AI systems capable of supporting these management functions must operate across multiple interconnected layers.

### 4.1. Layered Architecture Model

Based on the articles included in the review, our expertise and the technology used in the rehabilitation domain, we conceptualize AI-driven rehabilitation management as a six-layer architecture:

(1) Data acquisition layer. This foundational layer agThisgates multimodal data from:

- Wearable motion sensors (IMUs, accelerometers, motion sensors).
- Physiological monitors (heart rate, ECG, oxygen saturation, EMG).
- Robotic devices and exoskeletons.
- Virtual reality interaction logs.
- Electronic health records (EHRs).
- Patient-reported outcome measures (PROMs).

Data fusion at this level enables longitudinal modeling of recovery trajectories.

(2) Patient phenotyping and stratification layer. Using supervised and unsupervised machine learning techniques, this layer identifies clinically meaningful patient subgroups based on:

- Functional baseline status.
- Comorbidity profiles.
- Neuroimaging biomarkers.
- Behavioral engagement patterns.

Clustering algorithms, dimensionality reduction models, and deep neural networks support risk stratification and prognosis estimation. This stage enables precision rehabilitation by tailoring therapy intensity and modality selection to predicted recovery potential.

(3) Therapy planning and optimization layer. At this level, AI moves from descriptive analytics to prescriptive decision support. Key algorithmic paradigms include:

- Predictive modeling for outcome forecasting.
- Reinforcement learning for adaptive therapy dosing.
- Constraint optimization for session scheduling.
- Bayesian updating for dynamic plan revision.

Rather than statically assigning exercises, the system continuously updates therapeutic dosage, intensity, and modality selection based on the observed performance and predicted response. This layer represents the core management function of architecture.

(4) Real-time monitoring and closed-loop adaptation layer. This operational layer enables continuous feedback control. Computer vision systems, time-series models, and anomaly detection algorithms monitor:

- Movement quality.
- Cardiovascular response.
- Adherence levels.
- Fatigue indicators.

Closed-loop adjustment mechanisms modify exercise difficulty, robotic assistance levels, or VR task complexity in real time. Importantly, this distinguishes AI-enabled management from traditional fixed rehabilitation protocols.

(5) Outcome evaluation and prognostic modeling layer. AI systems at this level evaluate program effectiveness using:

- Functional outcome metrics.
- Cognitive performance scores.
- Digital biomarkers are derived from wearables.
- Time-to-event modeling for complication risk.

Longitudinal learning models (e.g., recurrent neural networks, survival analysis models) estimate recovery trajectories and identify early deviations from expected progress. This layer transforms rehabilitation from reactive monitoring to predictive management.

(6) Resource allocation and system-level coordination layer. Beyond patient-level optimization, AI may operate on an institutional scale by:

- Optimizing therapist allocation.
- Prioritizing high-risk patients.
- Scheduling equipment uses.
- Forecasting demand for rehabilitation services.

Operations research algorithms and predictive modeling can improve the model’s cost-effectiveness and accessibility, addressing healthcare system constraints. This layer extends AI from clinical support to healthcare system orchestration.

### 4.2. Distinguishing Levels of AI in Rehabilitation

To clarify the conceptual ambiguity in the current literature (see Table 2), it is essential to differentiate four operational levels of AI:

✓ Device-Level AI: Intelligence embedded within a specific robot, exoskeleton, or VR module.
✓ Session-Level AI: Real-time adaptation of exercise parameters during a therapy session.
✓ Program-Level AI: Longitudinal optimization of a patient’s rehabilitation trajectory across weeks or months.
✓ System-Level AI: Institutional optimization across multiple patients and providers.

Most existing studies focus on device- and session-level intelligence. However, true rehabilitation management requires integration at the program and system levels. This multi-level distinction highlights the principal gap in the current literature and provides a framework for future research.

### 4.3. Closed-Loop Governance and Human Oversight

AI-driven rehabilitation management must incorporate clinician-in-the-loop governance mechanisms. Decision transparency, the explainability of predictive models, and override capabilities are critical for:

- Maintaining patient safety.
- Preventing algorithmic bias.
- Preserving therapeutic empathy.
- Ensuring regulatory compliance.

Hybrid intelligence, combining AI-driven recommendations with expert clinical supervision, represents the most realistic and ethically sustainable model of implementation [50].

### 4.4. From Technology Integration to Program Orchestration

Current rehabilitation research often evaluates isolated technologies such as robotic gait systems or virtual reality platforms. However, these tools become transformative only when integrated within structured management architecture [2,48].

The proposed framework reframes artificial intelligence not as a collection of tools, but as an orchestration engine coordinating data, decision-making, adaptation, and evaluation across the rehabilitation continuum.

By articulating this layered architecture, the present review moves beyond descriptive technology summaries and establishes a systems-engineering perspective for AI-enabled rehabilitation management.

## 5. Evaluation and Translational Considerations

The integration of artificial intelligence (AI) in the planning and management of medical rehabilitation programs is revolutionizing the field, providing enhanced outcomes across various conditions.

AI enables the customization of rehabilitation programs tailored to individual patient needs. Through machine learning algorithms and data analysis, AI can develop personalized treatment plans that adjust in real time based on patient progress and responses.

To move beyond a technology-centric description of artificial intelligence in rehabilitation, AI applications can be systematically organized according to their functional role within program management (see Table 3). Rather than focusing on devices such as robots, virtual reality systems, or wearables, a management-oriented framework clarifies how AI contributes to decision-making across the rehabilitation continuum.

1. Patient phenotyping and stratification

At the foundational decision-support level, AI systems perform patient phenotyping through supervised and unsupervised learning approaches, including clustering algorithms and deep neural networks. These methods process heterogeneous clinical data, such as neuroimaging, gait kinematics, physiological signals, and baseline functional scores, to identify meaningful subgroups within rehabilitation populations. The resulting output is a structured risk profile or recovery potential classification. This stratification enables precision rehabilitation by aligning therapy intensity, modality selection, and monitoring strategies with predicted recovery trajectories.

2. Therapy optimization and prescriptive planning

Beyond descriptive analytics, AI advances into prescriptive decision-making through reinforcement learning, Bayesian optimization, and regression-based modeling. Using performance metrics, fatigue indicators, adherence patterns, and historical response data, these systems dynamically adjust therapy dosage and intensity. The decision output consists of an optimized therapeutic trajectory rather than a static prescription. This adaptive planning function represents the core of program-level intelligence, as it enables longitudinal refinement of rehabilitation strategies based on ongoing feedback.

3. Progress prediction and prognostic modeling

AI-driven time-series models, including recurrent neural networks and other longitudinal learning architectures, support predictive analytics within rehabilitation management. By analyzing continuous performance data and digital biomarkers, these systems generate individualized prognosis curves and early-warning indicators for stagnation or complication risk. The ability to forecast recovery trajectories transforms rehabilitation from reactive monitoring into predictive management, supporting timely intervention adjustments and outcome optimization [51].

4. Resource allocation and system-level coordination

At the institutional level, optimization algorithms and operations research models extend AI from patient-centered analytics to system-level orchestration. By incorporating institutional constraints, such as therapist availability, equipment usage, and patient load, AI systems generate scheduling plans and resource distribution strategies. This function enhances operational efficiency and scalability, addressing workforce limitations and healthcare system pressures. Importantly, system-level intelligence requires human oversight and governance mechanisms to ensure alignment with clinical priorities and ethical standards.

**Figure 3:**
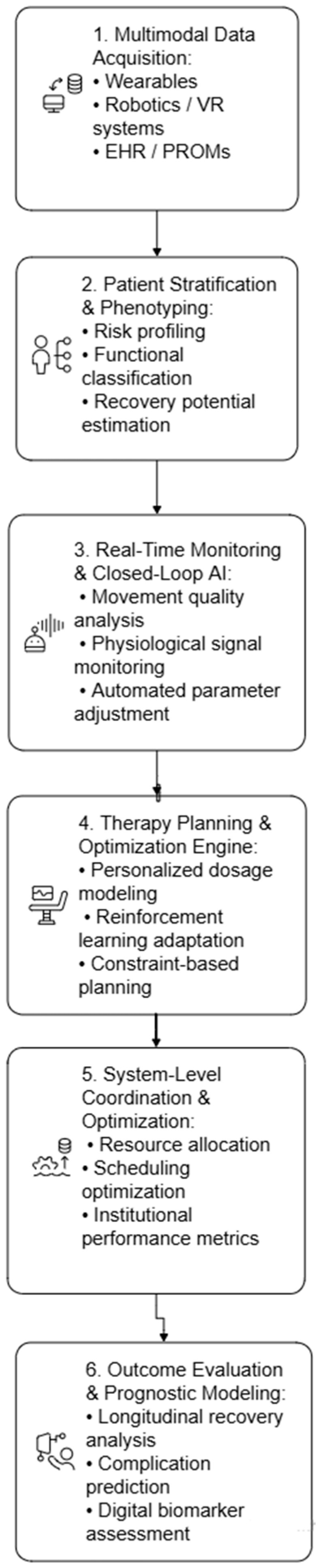
Layered system architecture for AI-driven rehabilitation program management.

AI architectures in rehabilitation (see Figure 3) typically encompass machine learning models, neural networks, and intelligent control systems. For instance, soft exoskeletons utilize intelligent control algorithms based on AI and machine learning to adapt to a user’s gait, posture, and terrain, providing a seamless and natural walking experience for individuals with mobility impairments [19].

Figure 3 illustrates the proposed layered architecture of AI-driven rehabilitation program management. The framework is organized into six interconnected layers that progressively transform raw patient data into adaptive, longitudinal rehabilitation strategies.

At the base, the Multimodal Data Acquisition Layer integrates heterogeneous data streams, including wearable sensor outputs, robotic device telemetry, virtual reality interaction logs, physiological signals, and electronic health records. These data are structured and preprocessed for downstream modeling.

The patient phenotyping and stratification layer applies machine learning techniques, such as clustering algorithms and deep neural networks, to classify patients into risk-adjusted recovery profiles. This layer supports precision rehabilitation by identifying functional baselines and predicting recovery potential.

The therapy planning and optimization layer constitutes the central decision-making component. Here, predictive analytics, reinforcement learning, and constraint-based optimization algorithms generate individualized therapy plans. These plans are not static prescriptions, but dynamic trajectories continuously refined through performance feedback.

The real-time monitoring and closed-loop adaptation layer enables ongoing calibration of therapy parameters. Time-series models and computer vision systems detect deviations from expected performance and trigger automated adjustments in exercise intensity, robotic assistance, or virtual task complexity [39].

Above this, the outcome evaluation and prognostic modeling layer assesses longitudinal recovery trajectories using survival analysis, recurrent neural networks, and digital biomarker analytics. This layer supports early detection of stagnation or complication risk.

At the highest level, the system-level coordination and resource optimization layer extend AI functionality to institutional management, including therapist allocation, scheduling optimization, and equipment utilization forecasting.

A horizontal clinician oversight and governance layer span all levels, ensuring transparency, explainability, ethical compliance, and human validation of AI-generated decisions.

Organizing AI applications according to management functions clarifies the progression from descriptive analytics (phenotyping) to prescriptive decision support (therapy optimization), predictive modeling (progress forecasting), and ultimately institutional coordination (resource allocation). This structured perspective distinguishes device-level automation from true program-level and system-level management intelligence.

To clarify the distinction between isolated AI applications and program-level management capabilities, we synthesized the literature according to the functional layers of rehabilitation management. Table 4 presents a comparative framework that evaluates AI technologies across algorithmic paradigms, data dependencies, decision scope, and translational maturity.

In summary, AI is transforming the landscape of medical rehabilitation by providing personalized, efficient, and effective therapeutic interventions. Its ability to analyze complex data, predict outcomes, and adapt treatments in real-time makes it an invaluable tool in improving patient care and recovery outcomes across various medical conditions. AI-driven rehabilitation programs are going to become more advanced and accessible, making them a vital part of modern healthcare.

## 6. Discussion

This review demonstrates that artificial intelligence in rehabilitation has progressed substantially at the level of device augmentation and session-based adaptation yet remains limited in its capacity to orchestrate full rehabilitation programs. Across the 61 included studies, a consistent pattern emerges when evidence is examined at the level of individual domains and methodological approaches. Studies focusing on robotics and wearable systems [11,16,18,19] provide the strongest technical validation, particularly in adaptive control and real-time biomechanical feedback. Similarly, studies investigating virtual and extended reality systems [13,14,26,30,31,32,33] consistently report improvements in engagement and functional outcomes, although these effects are primarily demonstrated in controlled or short-term settings. In contrast, studies addressing program-level management functions, such as longitudinal therapy optimization and institutional coordination [22,32,42,43,44,45], remain fewer in number and are largely limited to pilot studies or early-stage implementations.

**Figure 4:**
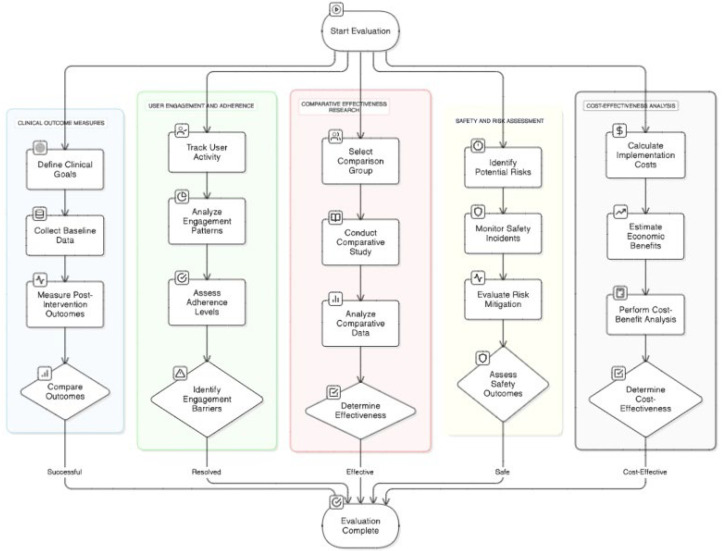
AI integration evaluation in rehabilitation programs.

To better contextualize these findings (see Figure 4), the evaluation of AI integration in rehabilitation programs can be mapped to the types of evidence reported across the included studies. Rather than representing abstract evaluation categories, the following dimensions are directly supported by specific groups of studies:

- Clinical outcome measures: several studies employ standardized clinical metrics to quantify functional and cognitive improvements. For example, neuropsychological assessment tools such as ANAM have been used to monitor cognitive changes over time in AI-assisted rehabilitation settings [52]. In addition, randomized and observational studies in digital rehabilitation platforms [10,14] demonstrate comparable or improved outcomes relative to conventional therapy, particularly in motor recovery and functional independence.
- User engagement and adherence: evidence from digital therapy platforms and AI-driven remote rehabilitation systems indicate consistently higher engagement rates compared to traditional care models. Studies evaluating commercial and clinical platforms, such as those reported by Sword Health [53,54], highlight increased adherence and sustained patient participation, particularly in home-based settings.
- Comparative effectiveness research: a subset of the included studies employs randomized controlled trial designs to compare AI-integrated interventions with standard rehabilitation approaches. For instance, VR-based therapeutic interventions demonstrate high effectiveness in specific applications, such as anxiety-related conditions and motor rehabilitation [55,56,57]. However, these studies are typically limited to specific modalities and do not yet extend to full program-level management.
- Safety and risk assessment: safety considerations are addressed primarily in studies involving socially assistive robotics and AI-supported monitoring systems [58,59]. These studies demonstrate that AI-enabled interventions can be implemented without compromising patient safety if monitoring and clinician oversight mechanisms are in place.
- Cost-effectiveness analysis: economic evaluations remain relatively limited but are addressed in studies focusing on digital and telerehabilitation systems [60,61]. These studies suggest that AI integration may reduce healthcare utilization and associated costs through improved adherence and reduced need for in-person interventions, although comprehensive cost-effectiveness analyses are still lacking.

By grounding these evaluation dimensions in the evidence provided by specific groups of studies, the analysis moves beyond a purely conceptual framework and reflects the actual methodological approaches observed in literature.

A central finding of this synthesis is the translational asymmetry across management layers, which becomes evident when studies are grouped according to their functional contribution. Device- and session-level AI applications, including adaptive exoskeleton control [11,16] and VR-based task modulation [13,26], are supported by multiple studies with measurable clinical benefits and moderate levels of validation. In contrast, program-level AI approaches, such as reinforcement learning-based therapy optimization [22] and remote monitoring architectures integrating multimodal data streams [32], are supported by a smaller number of studies, often with limited sample sizes and short follow-up durations. Similarly, system-level applications, including resource allocation and institutional coordination [42,43,44,45], are rarely validated in real-world clinical settings.

**Figure 5:**
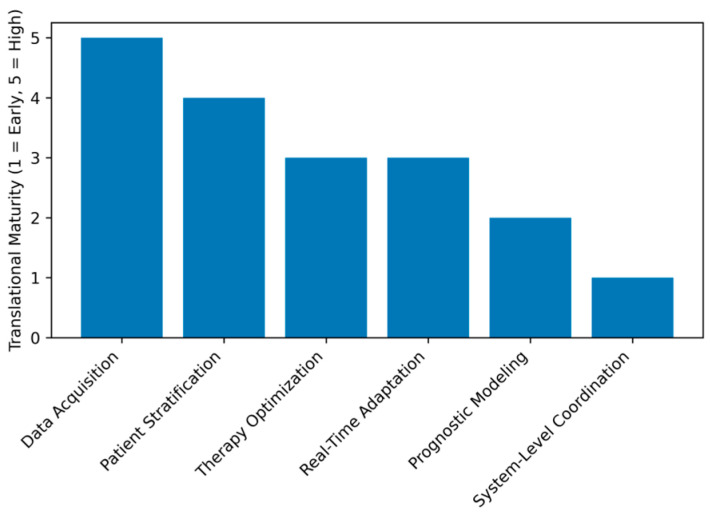
Translational maturity across AI rehabilitation management layers.

This imbalance reflects a structural limitation identified across the studies included as we can see in Figure 5. Most AI systems are designed and evaluated as isolated functional components, rather than as elements of an integrated rehabilitation management architecture.

Rehabilitation is inherently longitudinal and adaptive, requiring coordinated processes encompassing patient phenotyping, therapy dosage optimization, real-time performance monitoring, outcome forecasting, and resource allocation. The proposed six-layer architecture synthesizes these functions by integrating evidence from studies across different domains and methodological approaches. Importantly, this framework is not derived solely from conceptual reasoning, but from the observed distribution of functionalities across the reviewed studies, where each layer corresponds to a cluster of existing implementations.

Nevertheless, several barriers to integration are consistently reported across literature. Studies involving wearable systems and digital platforms highlight issues related to data heterogeneity and interoperability [12,18], while studies focusing on predictive modeling emphasize limitations in dataset size and external validation [22,24,41]. Furthermore, many studies rely on retrospective or single-center datasets, limiting generalizability. Reinforcement learning approaches and prognostic models, although promising, remain insufficiently validated in large, multicenter prospective trials.

Ethical and governance considerations are increasingly emphasized in studies addressing program-level and system-level AI. As decision-making shifts from device-level assistance to program-level optimization, issues such as algorithmic transparency, bias, and accountability become critical. Several studies highlight the importance of clinician-in-the-loop frameworks and explainability mechanisms to ensure safe and responsible implementation [50]. The integration of provable ethics and explainability frameworks, functioning as a trustworthy ethical firewall, represents an essential direction for future research.

From a health systems perspective, evidence from telerehabilitation and remote monitoring studies [42,43,44,45] suggests that AI-driven rehabilitation may improve efficiency through optimized scheduling, personalized therapy trajectories, and continuous monitoring. However, the economic impact of these systems remains insufficiently quantified, and the existing studies provide only preliminary insights into cost-effectiveness.

Future research should therefore focus on:

- Longitudinal, multicenter validation studies.
- Standardized outcome reporting across rehabilitation domains.
- The development of interoperable data infrastructures.
- The integration of AI systems across multiple management layers.

In summary, the field is transitioning from isolated technological enhancements toward structured, AI-enabled rehabilitation management ecosystems. By explicitly linking the findings to the underlying evidence base, this review highlights both the progress achieved and the critical gaps that remain. The architectural perspective advanced in this work provides a framework for advancing artificial intelligence from fragmented innovation to coordinated, system-level rehabilitation program management.

## 7. Conclusions

Artificial intelligence has already demonstrated measurable value in specific rehabilitation applications, including robotic assistance, motion analysis, predictive modeling, and telerehabilitation platforms. However, the central challenge facing the field is no longer whether AI can support isolated therapeutic tasks, but how these technologies can be coherently integrated into structured rehabilitation program management systems.

This review reframed AI in rehabilitation from a collection of technological tools to a multi-layer orchestration architecture encompassing data integration, patient stratification, therapy optimization, real-time adaptation, longitudinal prognostic modeling, and institutional resource coordination. By synthesizing empirical studies through a management-layer perspective, we identified a clear asymmetry in the literature: most AI implementations operate at device- or session-level intelligence, while true program-level and system-level coordination remains underdeveloped.

The transition from assistive intelligence to managerial intelligence represents the next critical evolution of the field. Reinforcement learning-based dosage optimization, longitudinal recovery modeling, digital biomarkers derived from continuous monitoring, and federated data infrastructures offer promising directions for enabling scalable and adaptive rehabilitation ecosystems. Yet, significant structural barriers persist. These include limited dataset generalizability, the lack of interoperability between AI systems and electronic health records, insufficient prospective clinical validation, and the need for explainable, clinician-in-the-loop governance mechanisms.

Furthermore, ethical considerations, particularly algorithmic bias, transparency, accountability, and preservation of therapeutic human interaction, must be embedded within future system designs rather than treated as secondary constraints. The advancement of provable AI ethics and explainability frameworks in medical and educational AI agents, functioning as a trustworthy ethical firewall, will be essential for ensuring that automated decision-support systems remain auditable, bias-aware, and aligned with clinical responsibility. Embedding formal verification mechanisms, transparent model interpretability, and enforceable ethical constraints into AI architectures will help safeguard patient trust and regulatory compliance.

Sustainable implementation will require interdisciplinary collaboration among biomedical engineers, rehabilitation clinicians, data scientists, and health system administrators. Ethical oversight, regulatory alignment, and human-centered system design must evolve in parallel with technical innovation.

In conclusion, the future of AI in medical rehabilitation lies not in expanding the number of intelligent devices, but in constructing integrated, adaptive, ethically grounded, and accountable management architectures capable of orchestrating the entire rehabilitation trajectory. Establishing standardized frameworks for program-level AI integration, validating them through longitudinal multicenter trials, and embedding provable ethical safeguards within their design will be essential to translating artificial intelligence from promising innovation to reliable clinical infrastructure.

By advancing a system-oriented and ethically structured perspective, this work contributes a conceptual foundation for the next generation of trustworthy intelligent rehabilitation management platforms.

## Figures and Tables

**Table 1:** Management role of AI integration in rehabilitation domains.

Domain	AI Integration Type	Management Role	Clinical Validation Level	Deployment Maturity	Key Limitations
Robotics and Exoskeletons	Adaptive control; ML gait analysis	Session-level adaptation	Moderate high (RCTs exist)	Moderate	Limited interoperability
Wearable Monitoring Systems	Signal processing; real-time analytics	Monitoring + feedback	High (technical validation)	High (device-level)	Data standardization
VR/XR Systems	Adaptive task generation; engagement analytics	Session-level personalization	Moderate	Moderate	Limited longitudinal integration
Neurological AI Platforms	Neuroimaging ML; risk stratification	Diagnostics + planning	Growing	Emerging	Small datasets
Neurodegenerative Prediction	Prognostic ML; cognitive AI training	Predictive management	Moderate	Emerging	Short follow-up
Cardiovascular Telerehabilitation	Remote ML monitoring; risk prediction	Monitoring + adherence	Moderate	Emerging	Heterogeneity of protocols

**Table 2:** Differentiation of AI levels in rehabilitation management.

Intelligence Level	Scope	Example Application	Temporal Horizon	Decision Autonomy	Integration Complexity
Device-Level AI	Single tool	Exoskeleton gait adjustment	Seconds	High (local)	Low
Session-Level AI	One therapy session	VR difficulty modulation	Minutes–hours	Semi-autonomous	Moderate
Program-Level AI	Multi-week therapy planning	Adaptive dosage redesign	Weeks–months	Decision-support dominant	High
System-Level AI	Institutional	Patient triage; resource planning	Months–years	Low (human-in-loop required)	Very High

**Table 3:** Structured AI management architecture.

Management Function	AI Techniques	Data Input	Decision Output
Patient phenotyping	clustering, deep learning	imaging, gait data	risk profile
Therapy optimization	reinforcement learning	performance metrics	dosage/intensity
Progress prediction	time-series ML	longitudinal data	prognosis curve
Resource allocation	optimization algorithms	institutional constraints	scheduling plan

**Table 4:** Comparative analysis of AI applications in rehabilitation across management layers, algorithmic paradigms, decision scope, and translational maturity.

Management Layer	AI Function	Typical Algorithms Used	Data Inputs	Decision Output	Evidence Level	Key Limitations	Translational Maturity
Data Acquisition and Integration [11,18,46]	Multimodal sensor fusion	Signal processing, CNNs, sensor fusion models	IMUs, ECG, VR logs, EHR	Structured patient dataset	High (technical validation studies)	Interoperability issues, data heterogeneity	High at device level
Patient Stratification [7,21,23]	Risk profiling; phenotyping	Clustering (k-means), SVM, deep neural networks	Baseline functional scores, imaging, demographics	Recovery probability, risk classification	Moderate high	Limited external validation, dataset bias	Moderate
Therapy Planning and Optimization [2,10,38]	Exercise selection; intensity prescription	Reinforcement learning, Bayesian optimization, regression models	Historical response data, fatigue metrics, performance trends	Individualized therapy trajectory	Moderate	Few large RCTs; limited explainability	Emerging
Real-Time Adaptation [11,20,23]	Closed-loop adjustment	Time-series models, LSTM, anomaly detection, computer vision, pose estimation	Live movement data, cardiovascular signals	Dynamic difficulty adjustment, robotic assistance modulation	Moderate	Mostly device-level adaptation, weak cross-platform integration	Moderate
Outcome and Prognostic Modeling [34,35,37]	Recovery forecasting; complication prediction	Survival analysis, recurrent neural networks, ensemble ML	Longitudinal functional data, biomarkers	Predicted trajectory, early risk alerts	Growing but variable	Small datasets, short follow-up durations	Emerging
System-Level Coordination [2,42,43]	Resource allocation; scheduling optimization	Linear programming, predictive analytics, optimization algorithms	Patient load, therapist availability, equipment uses	Optimized scheduling, workload distribution	Low (limited real-world deployment studies)	Rarely integrated with clinical AI systems	Early-stage

## Data Availability

The original contributions presented in this study are included in the article. Further inquiries can be directed to the corresponding author.

## Supplementary Materials

The following supporting information can be downloaded at: https://www.mdpi.com/article/10.3390/bioengineering13050539/s1. Included studies table auto extracted from the manuscript reference list provides a structured summary of all studies included in the qualitative synthesis, based on predefined data extraction variables, including rehabilitation domain, management layer, artificial intelligence (AI) methodology, validation level, and reported effectiveness.
